# Weaning indexes do not predict success with non invasive mechanical ventilation in extubation failure

**DOI:** 10.1186/2197-425X-3-S1-A177

**Published:** 2015-10-01

**Authors:** O Torres, L Delgado, E Monares, A Sanchez-Calzada, R Gastelum, JL Navarro, P Romano, J Franco

**Affiliations:** ABC Medical Center, Intensive Care, Mexico, Mexico; Hospital san Angel Inn Universidad, Intensive Care, Mexico, Mexico

## Introduction

Lung ultrasound (LUS) is a useful tool in the intensive care unit (ICU) and in mechanical ventilation it has been possible to measure lung collapse that presents after a spontaneous breathing test by measuring lung aeration loss and detecting those patients that probably will fail extubation and assessing the necessity of non invasive mechanical ventilation (NIMV) as well as those patients that fail the spontaneous breathing test as a weaning method. There is controversy whether the NIMV can help in weaning in patients other than those with chronic obstructive pulmonary disease (COPD).

## Objectives

To determine those patients that will fail the use of NIMV by using LUS.

## Methods

Prospective observational study completed from April 2013 to April 2015. LUS were performed at minute 1 and 30 during a spontaneous ventilation test at PEEP of 0cmH2O and support pressure of 7cmH2O. Indexes to evaluate extubation were measured including negative inspiratory force, P01, superficial respiration index, also LUS in the 6 quadrants from left and right hemithorax evaluating the lines patterns determining if an A, B or AB pattern exist at minute 1 and 30. Successful weaning was defined as those patients that remained extubated for at least 72 hours.

## Results

A total of 79 patients were analyzed with 37 (46,7%) female population and a median age of 64 year (min. 19 max. 88 years). Means of length of stay in the ICU of 16 days, Charlson index 5,54 (min. 0 max. 17,3), SOFA score at admission 7 (min. 0 max. 7) and mean SOFA before extubation of 2. Of the 79 patients 88,6% (n = 70) had a successful extubation, 9 required NIMV for extubation failure treatment and 19 used NIMV as a weaning method. Of the 79 patients 17 (21,5%) presented extubation failure, the causes of failure were 10/79 for cardiac cause, 5/79 for respiratory cause and 2/79 for mixed cause. For this study only the patients that used NIMV after extubation were included (n = 28) and divided in those who were successfully extubated (57,1%) and those who fail extubation (42,9%). This group presented a mean age of 71,21 ± 10,69, with 13 (46,4%) women, Charlson index of 5,43( ± 2,39), SOFA 9( ± 3,43), VRS at minute 1 of 50,64( ± 16,74), VRS at minute 30 of 48,68 ( ± 24,16), P01 at minute 1 of 3( ± 1,96), P01 at minute 30 of 2,21( ± 0,99). Distribution between LUS patterns at minute 1 and 30 were respectively A: 10(35,7%) and 7 (25%), B: 10 (35,7%) in both times and AB: 8 (28,6%) and 12 (39,3%). Also we found that 42,9% of those patients with NIMV as a weaning method or as a extubation failure treatment had to be reintubated. There were no significant differences between the two groups in the variables measured.

## Conclusions

None of the indexes employed predicts the success of NIMV for extubation failure treatment. Further investigation is needed to develop new indexes that can predict success of NIMV as a treatment for extubation failure.

